# Obituary

**Published:** 1883-04

**Authors:** 


### Obituary.

DR. GEORGE M. BEARD, the well-known physician and specialist in hypnotism and insanity, died January 23d in New York City after a short illness. Dr. BEARD was born in Connecticut May 8th, 1839. He graduated at Yale in 1862, and at the College of Physicians and Surgeons in 1866. Dr. BEARD was a voluminous writer, and during the latter years of his life gave his attention to hypnotism, or mesmerism. An article on trance and trancoidal states in the lower animals from his pen will be found in No. 2 of Vol. II. of this Journal.

JOHN STACK, a Veterinary Surgeon, residing at Orange, New Jersey, was instantly killed, being struck by a locomotive.

THE deaths of Dr. CAMARERO, Director of the Veterinary School of Leon, Spain, and Dr. FR. LUNDBERG, director of the Stockholm Veterinary School, is announced.

